# Supplementary material for: Dual Bmp-negative feedback loops modulate function of both AER and ZPA to buffer and constrain postaxial digit number
Source: Proc Natl Acad Sci U S A. 2025 Sep 23;122(39):e2427249122. doi: 10.1073/pnas.2427249122 (PMC12501167; doi:10.1073/pnas.2427249122)
Supplement: Supplementary file 1 — Appendix 01 (PDF) [file pnas.2427249122.sapp.pdf]

PNAS

Supporting Information for

Dual Bmp-negative feedback loops modulate function of both AER and ZPA to buffer and constrain postaxial digit number

Rashmi Patel and Susan Mackem

corresponding author: Susan Mackem

Email: mackems@mail.nih.gov

This PDF file includes:

Figures S1 to S9

Table S1

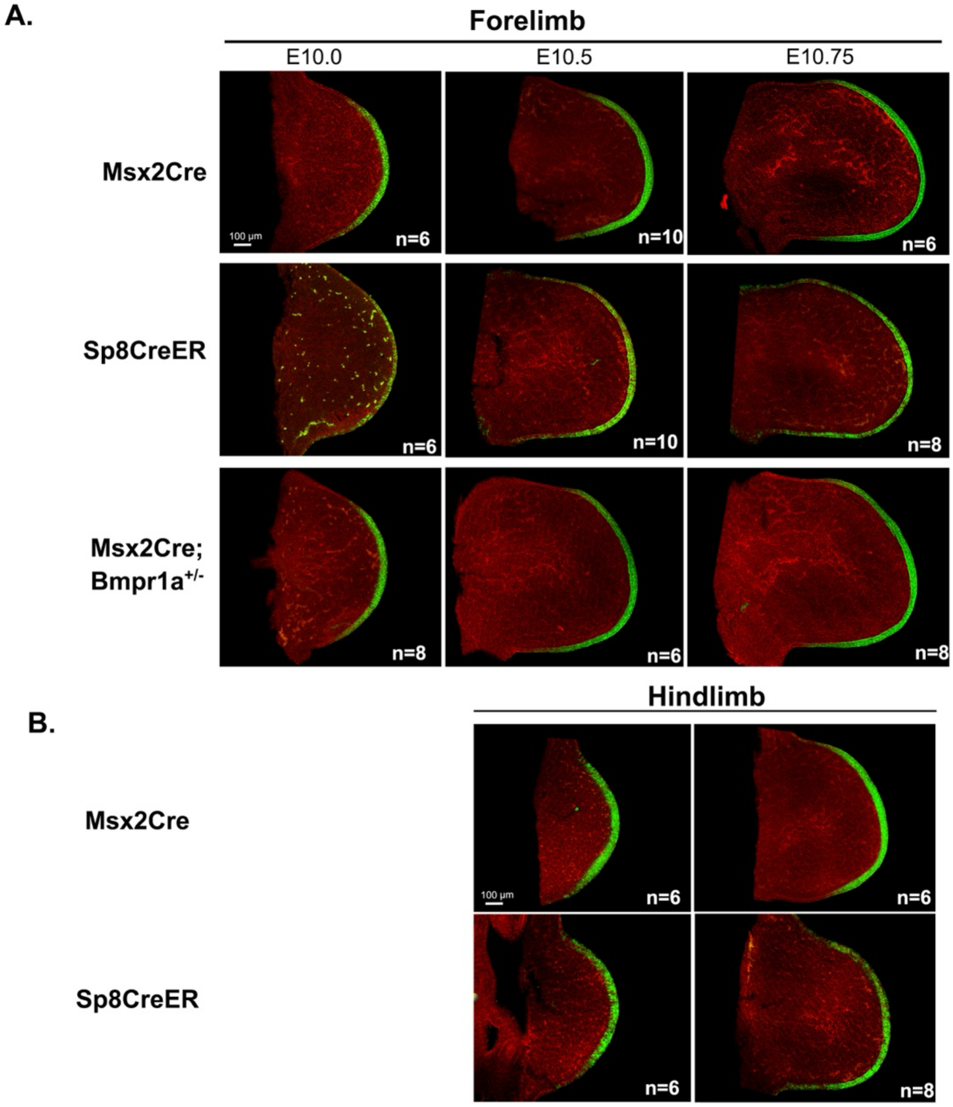

Fig. S1. Recombination dynamics of AER-specific Cre drivers used.

Msx2Cre and Sp8CreER recombination efficiency over time was checked using the Rosa-mT/mG reporter as described in Methods. In all panels, representative confocal images of a central 20um optical section are shown to illustrate both recombined (green signal, membrane-EGFP) and unrecombined cells (red signal, membrane-tdTomato) in AER ectoderm at the time points indicated (E10, E10.5, E10.75) in both Forelimb (A) and Hindlimb (B) buds. N, number of independent limb buds analyzed for that time point.

Msx2Cre was highly efficient, and largely complete at E10 in forelimb, and E10.5 in hindlimb. For Msx2Cre, the Msx2Cre;*Bmpr1a*$^{+/-}$ allele was used in experiments analyzing AER removal of *Bmpr1a,* and gave similarly efficient recombination.

Sp8CreER recombination was initially somewhat mosaic at E10, but largely complete by E10.5 in forelimb, probably reflecting later onset of Cre-activity. For all Sp8creER analyses, a single dose of 2mg tamoxifen was given at E9.5. The green signals present in mesoderm of the Sp8creER E10 Forelimb image are due to autofluorescence from red cells in capillaries.

In all figures, limb buds are oriented with anterior at top and distal border at right side of image panel and scale bar (100mm size) is shown once in first panel for a set of HCR images all at the same magnification.

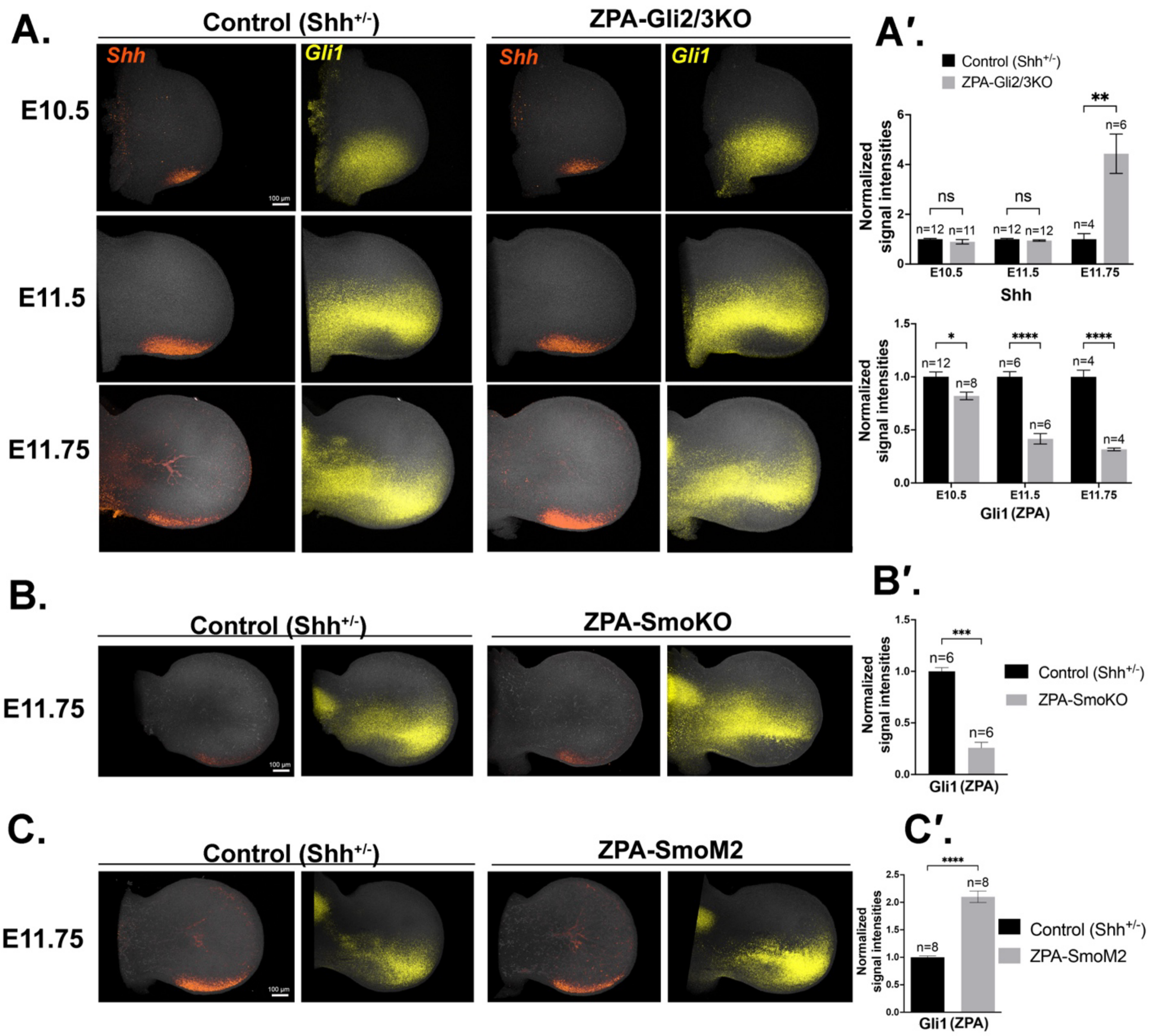

Fig. S2. Analysis of *Shh* and *Gli1* in ZPA confirms efficacy of ZPA-Gli2/3KO, ZPA-SmoKO and ZPA-SmoM2.

A. Simultaneous HCRs for *Shh* and *Gli1* (Shh-response) in ZPA regions at different stages indicated for ZPA-Gli2/3KO compared to sibling controls. Elevated *Shh* expression and loss of Shh-response (*Gli1*) within the ZPA by E11.5-E11.75.

A'. Bar graphs of HCR data show average signal intensities for *Shh* and for *Gli1* in the posterior ZPA region of ZPA-Gli2/3KO limb buds compared to sibling controls.

B. Simultaneous HCRs of *Shh* and *Gli1* expression in ZPA region of ZPA-SmoKO shows loss of Shh-response (*Gli1*) within the ZPA at E11.75.

B'. Bar graph of HCR data shows average signal intensities for *Gli1* in the posterior ZPA region of ZPA-SmoKO limb buds compared to sibling controls.

C. Simultaneous HCRs of *Shh* and *Gli1* expression in ZPA region of ZPA-SmoM2 shows increased Shh-response (*Gli1*) within the ZPA at E11.75.

C'. Bar graph of HCR data shows average signal intensities for *Gli1* in the posterior ZPA region of ZPA-SmoM2 limb buds compared to sibling controls. n, forelimb bud numbers analyzed for each genotype.

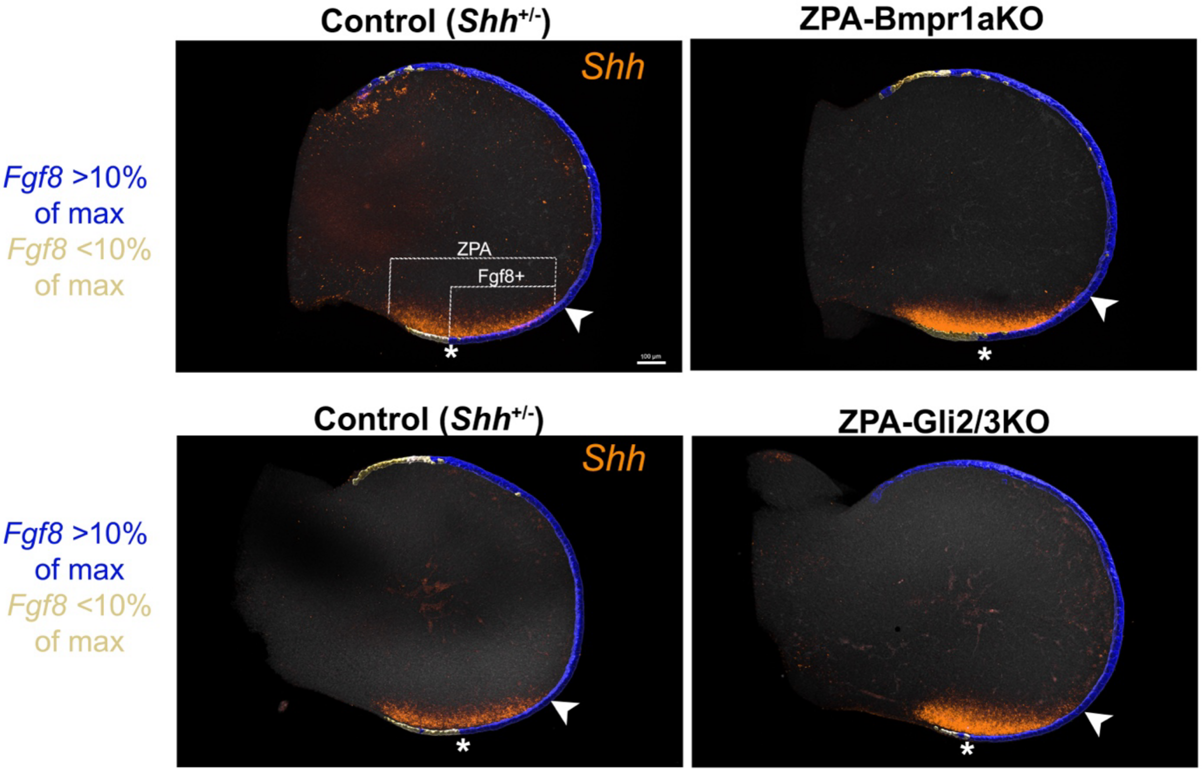

Fig. S3. Determination of ZPA-AER overlap extent using fluorescence quantitation by Imaris. Simultaneous HCRs of *Shh* and *Fgf8* expression were analyzed to determine extent of AER-ZPA overlap (white lines indicate Fgf8+ and total ZPA length) in different mutants (representative egs. shown for ZPA-Bmpr1aKO and ZPA-Gli2/3KO). Arrowheads mark point taken as the start of AER-ZPA overlap, where *Shh* expression ends distally. Imaris was used to computationally determine the relative *Fgf8* HCR intensities along the AER and the point at which expression drops to 10% or less of the maximum average *Fgf8* intensity for that limb bud was arbitrarily designated as the proximal limb bud endpoint of the AER overlap (*) with ZPA in all samples. The 10% cut-off is highlighted in the examples above, in which Imaris was used to assign blue color to AER above the 10% threshold, and gold color to AER regions below the 10% level. (see also Methods for details).

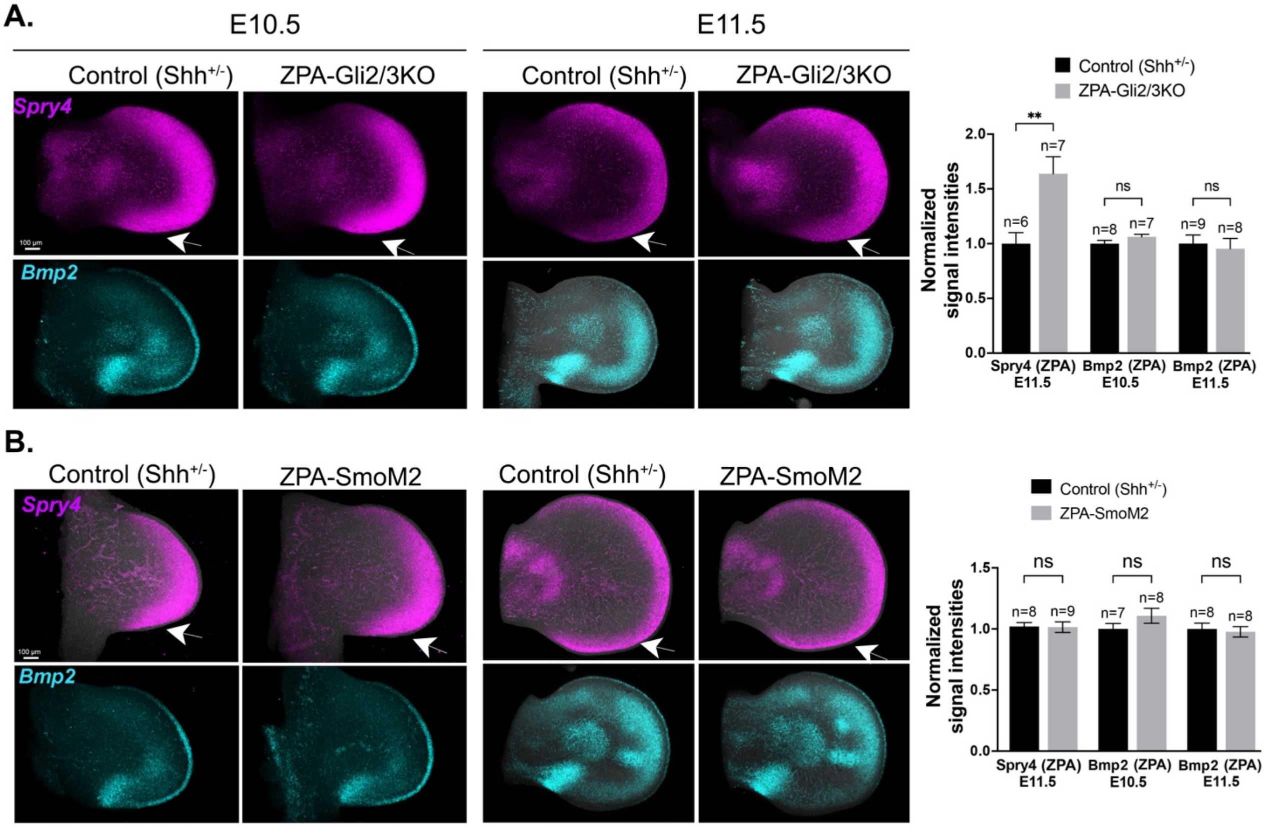

Fig. S4. Analysis of AER function (*Spry4*) and *Bmp2* expression in ZPA region of ZPA-Gli2/3KO and ZPA-SmoM2.

HCRs comparing *Spry4* and *Bmp2* in ZPA-Gli2/3KO (A) and ZPA-SmoM2 (B) E10.5-E11.5 limb buds with sibling controls.

A. *Spry4* is elevated in the ZPA-Gli2/3KO posterior limb bud ZPA region (arrows) in E11.5 limb buds compared to sibling controls, and *Bmp2* in posterior limb bud ZPA region is unchanged. Bar graphs of HCR data show average normalized signal intensities for *Spry4* and *Bmp2* in posterior ZPA region of ZPA-Gli2/3KO and controls. n, forelimb bud numbers analyzed.

B. Both *Spry4* and *Bmp2* are unchanged in the ZPA-SmoM2 posterior limb bud ZPA region (arrows) compared to sibling controls. Bar graphs of HCR data show average normalized signal intensities for *Spry4* and *Bmp2* in posterior ZPA region of ZPA-SmoM2 and controls. n, forelimb bud numbers analyzed.

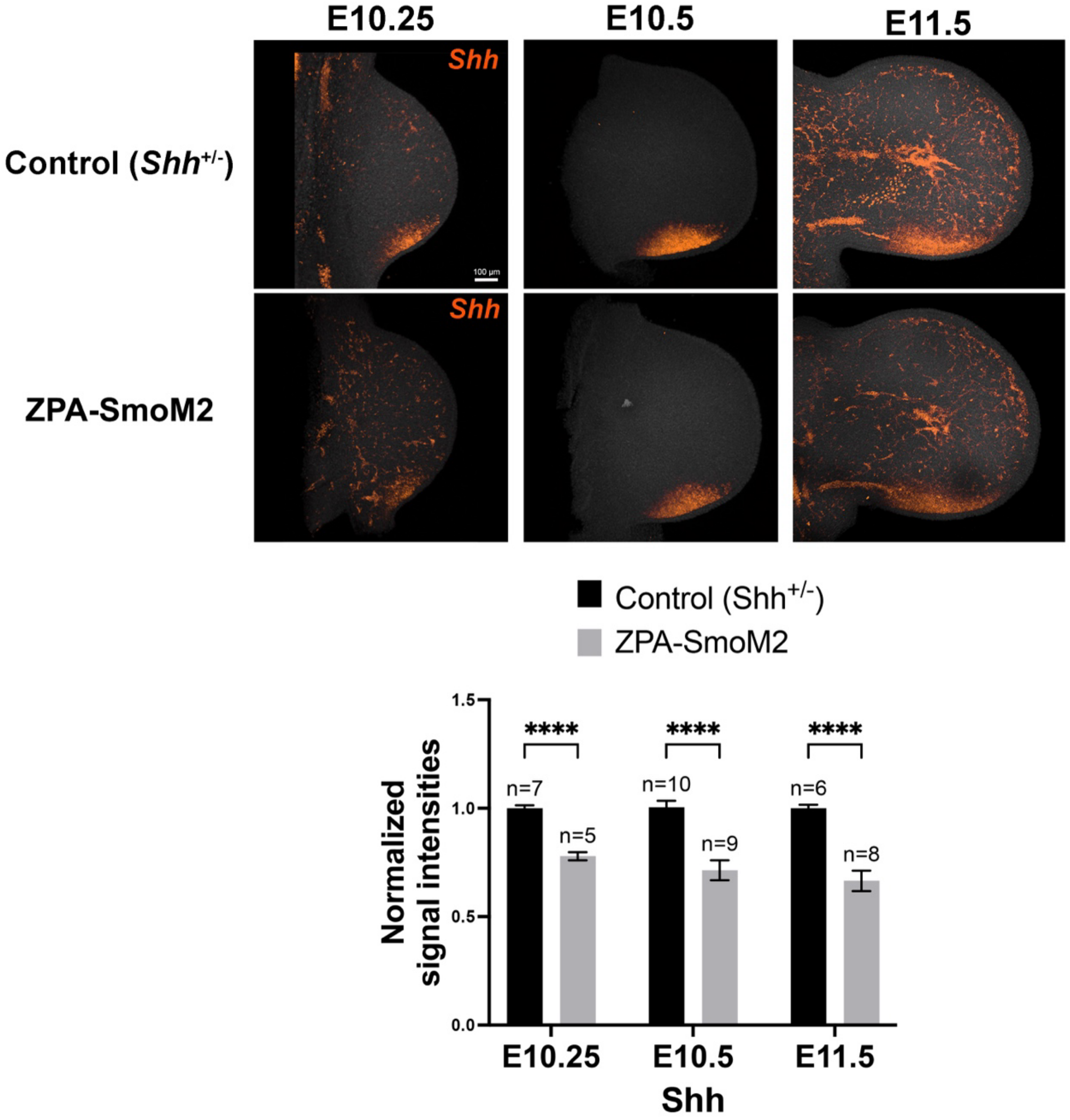

Fig. S5. Analysis of ZPA-Shh shows rapid reduction of Shh expression in ZPA-SmoM2 limb buds. HCRs for *Shh* at different stages indicated for ZPA-SmoM2 compared to sibling control forelimb buds. *Shh* expression is rapidly reduced following SmoM2 activation in ZPA (already evident by E10.25). Bar graph of HCR data below shows average *Shh* signal intensities in ZPA for ZPA-SmoM2 limb buds compared to controls at E10.25 - E11.5. n, forelimb bud numbers analyzed for each genotype at each stage.

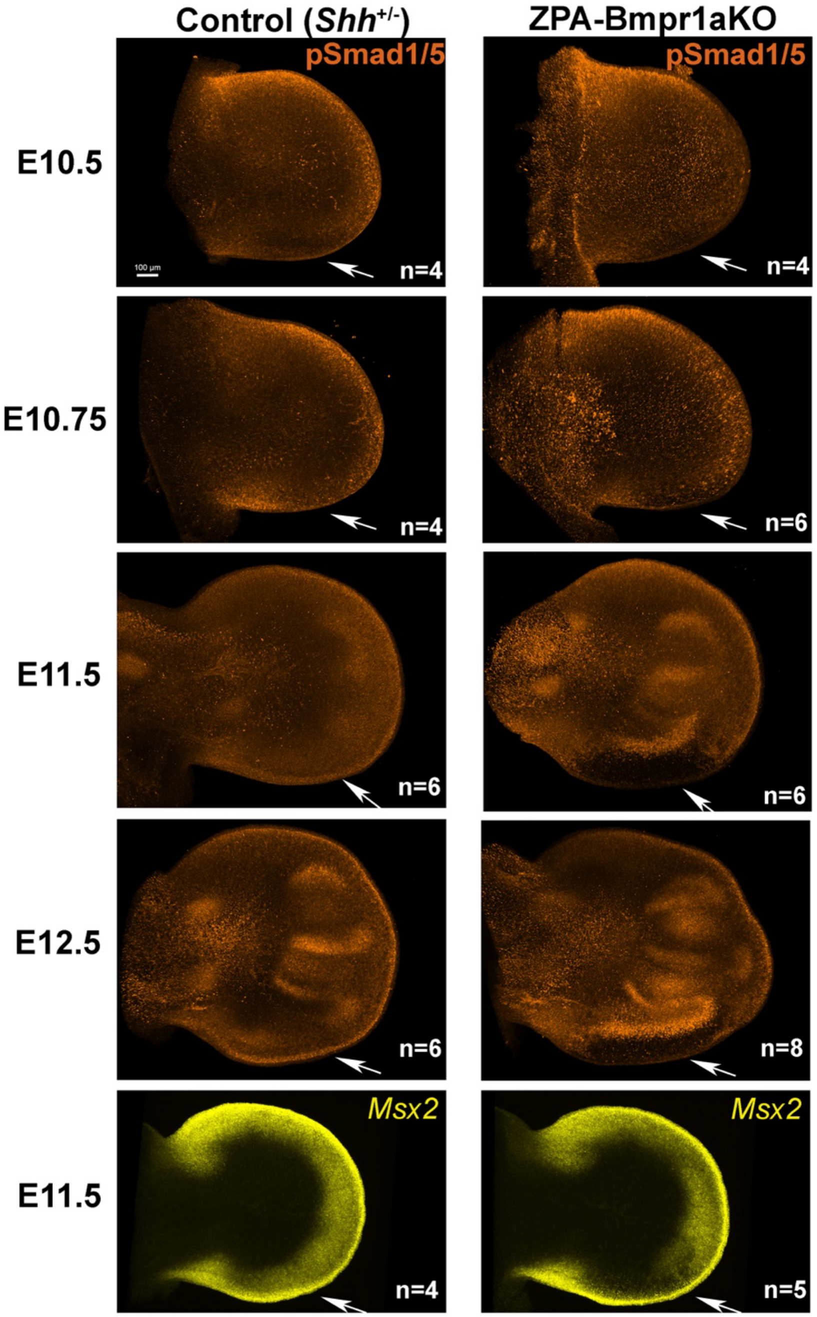

Fig. S6. Analysis of anti-pSmad1/5 levels confirms efficacy of ZPA-Bmpr1aKO by E11.5.
Whole mount anti-pSmad1/5 immunofluorescence staining in ZPA-Bmpr1aKO compared to sibling controls at different stages indicated (E10.5- E12.5) shows clear loss of pSmad1/5 by E11.5 in ZPA region (arrows). Bottom panels showing HCR for *Msx2* (Bmp-response reporter) also indicate loss of Bmp-response in ZPA region (arrows) at E11.5. n, forelimb bud numbers analyzed.

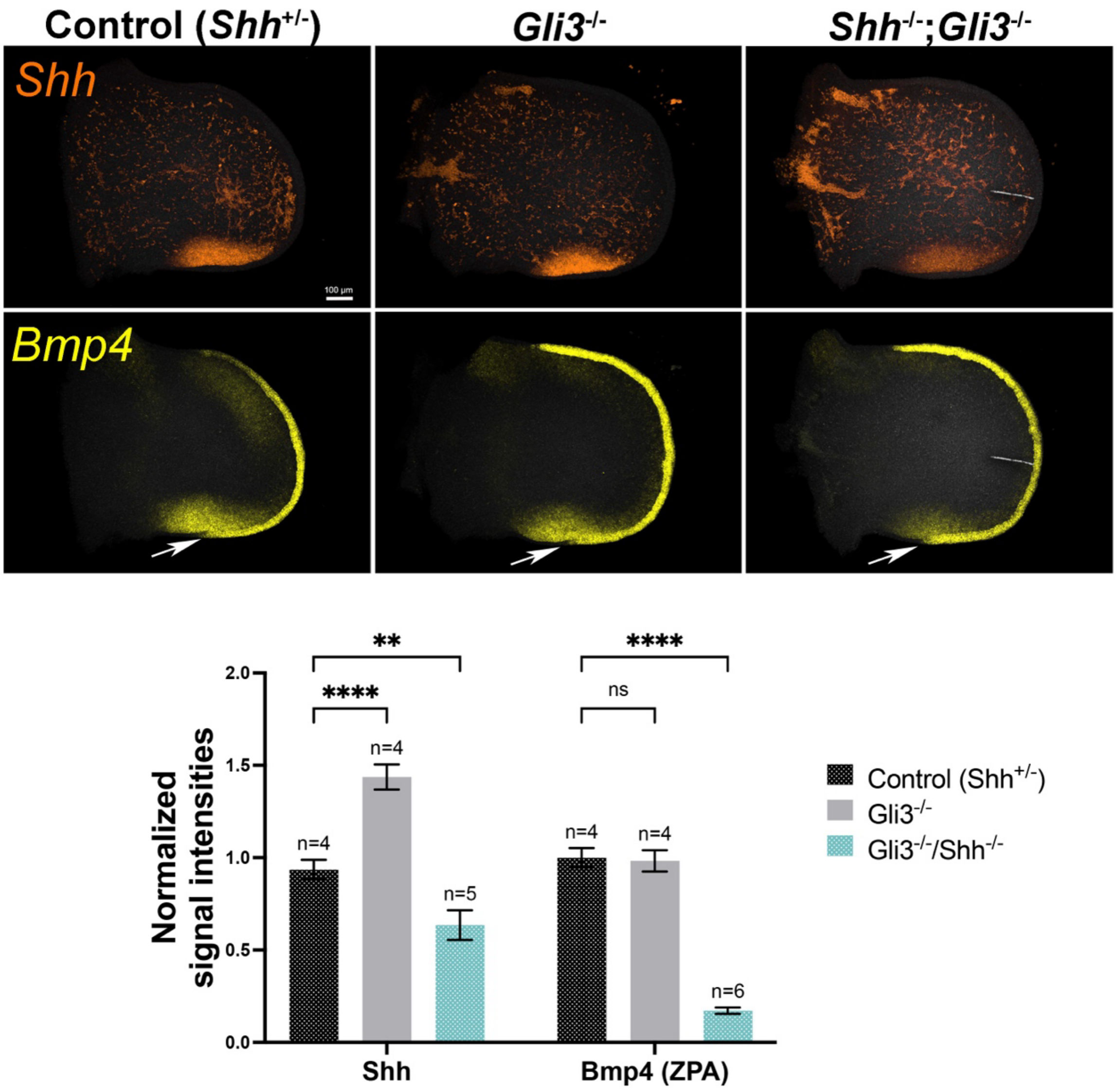

Fig. S7. ***Bmp4*** **is in part regulated by GliA.**

Simultaneous HCRs comparing *Bmp4* expression in posterior ZPA (*Shh*) region of *Gli3*$^{-/-}$ and *Shh*$^{-/-}$;*Gli3*$^{-/-}$ E10.5 forelimb buds with sibling controls. Loss of Gli3R (*Gli3*$^{-/-}$) has little effect on *Bmp4*, but loss of all GliA function in the double knockout (*Shh*$^{-/-}$;*Gli3*$^{-/-}$) results in a moderate decrease in *Bmp4* (arrows), as quantitated in bar graph below showing average normalized signal intensities.

n, forelimb bud numbers analyzed for each genotype.

Note that the *Shh* signal intensity is elevated in *Gli3*$^{-/-}$ relative to the control (*Shh*$^{+/-}$) because of higher *Shh* gene dosage in the *Gli3*$^{-/-}$ single mutant, and that *Shh* is still detected in the double knockout owing to high stability of *Shh* mutant transcripts.

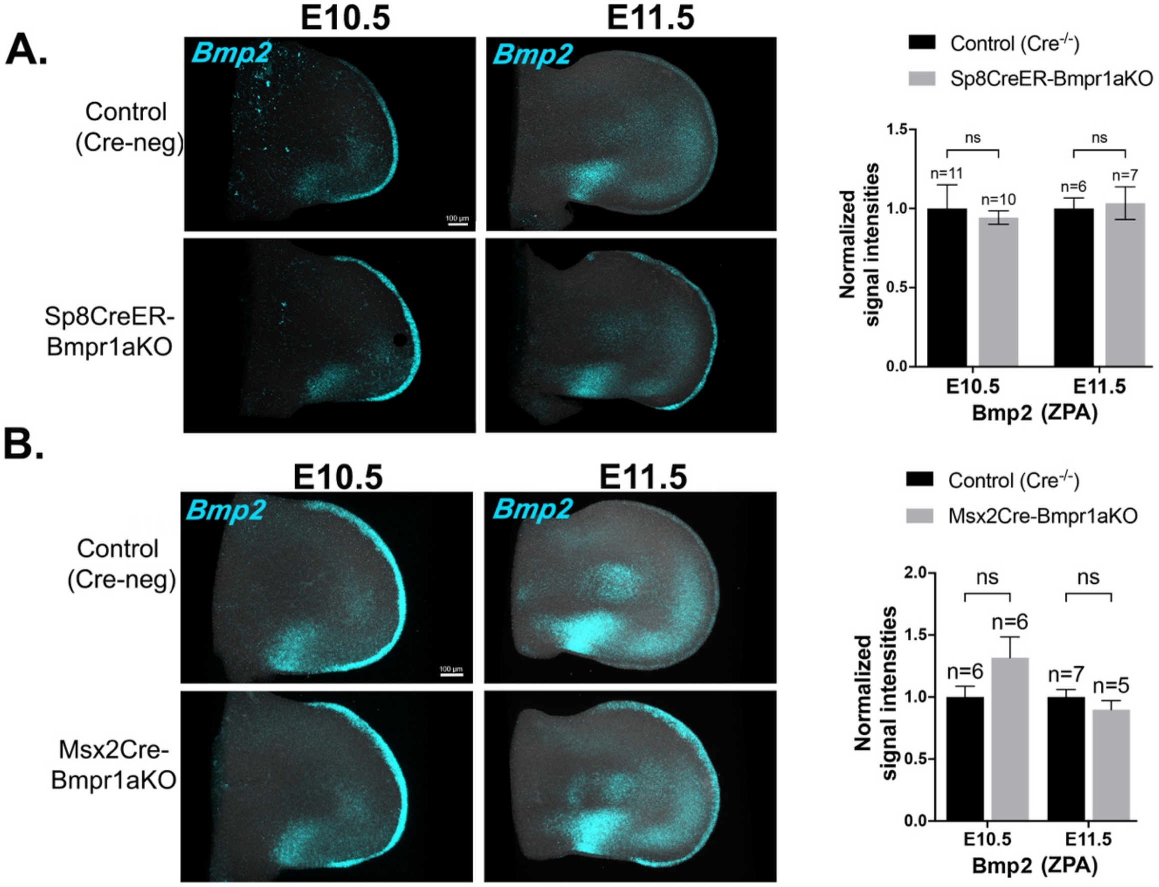

Fig. S8. ***Bmp2*** **in ZPA of both Sp8CreER-Bmpr1aKO and Msx2Cre-Bmpr1aKO is unchanged.**

A. HCRs at E10.5 and E11.5 show unchanged *Bmp2* expression in ZPA of Sp8CreER-Bmpr1aKO compared to sibling control forelimb buds, as indicated in bar graph to right showing average normalized signal intensities. (Tamoxifen given at E9.5).

B. HCRs at E10.5 and E11.5 show unchanged *Bmp2* expression in ZPA of Msx2Cre-Bmpr1aKO compared to sibling control forelimb buds, as indicated in bar graph to right showing average normalized signal intensities.

n, forelimb bud numbers analyzed for each genotype at each stage.

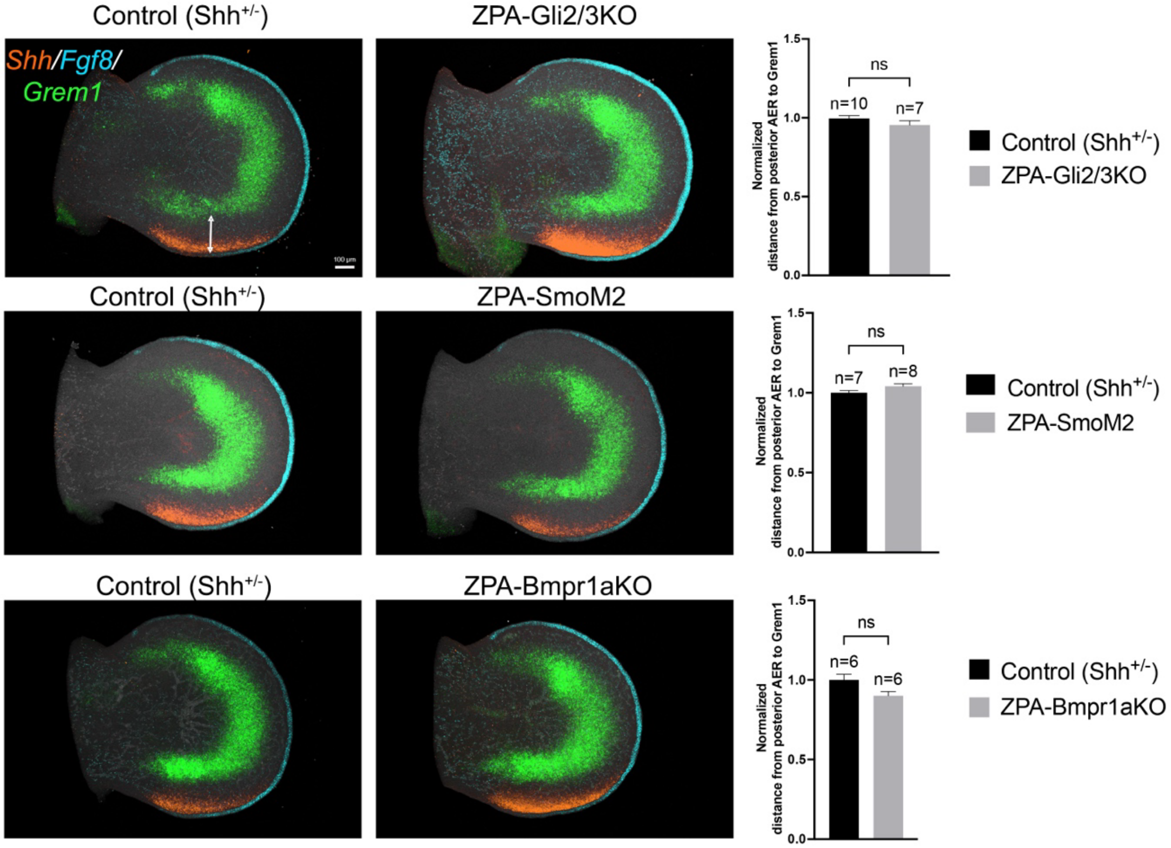

Fig. S9. No expansion of *Grem1* posterior domain into ZPA region occurs in ZPA-Gli2/3KO, ZPA-SmoM2 or ZPA-Bmpr1aKO.

Simultaneous HCRs for *Shh, Fgf8 and Grem1* at E11.5 for ZPA-Gli2/3KO, ZPA-SmoM2 and ZPA-Bmpr1aKO compared to sibling control forelimb buds. Relative distance between AER and posterior domain border of *Grem1* (white double headed arrow) was measured using Imaris for ZPA-Gli2/3KO, ZPA-SmoM2 and ZPA-Bmpr1aKO compared to sibling controls. Bar graphs to right show AER/*Fgf8*-to-*Grem1* border distance normalized to sibling control values. n, forelimb bud numbers analyzed for each genotype.

Table S1. Mutant and transgenic alleles used.

**Alleles crossed**			**Mutant genotype**	**Shorthand notation**
Shh$^{Cre/+}$	x	*Rosa*$^{SmoM2/ SmoM2}$	*Shh*$^{Cre/+}$; *Rosa*$^{SmoM2/+}$	ZPA-SmoM2
Shh$^{Cre/+}$;*Ptch1*$^{+/ Fl}$	x	*Ptch1*$^{Fl/Fl}$	*Shh*$^{Cre/+}$; *Ptch1*$^{Fl/Fl}$	ZPA-Ptch1KO
Shh$^{Cre/+}$; *Gli2*$^{+/Fl}$; *Gli3*$^{Fl/Fl}$	x	*Shh*$^{-/+}$; *Gli2*$^{Fl/Fl}$; *Gli3*$^{Fl/Fl}$	*Shh*$^{Cre/+}$; *Gli2*$^{Fl/Fl}$; *Gli3*$^{Fl/Fl}$	ZPA-Gli2/3KO
Shh$^{Cre/+}$; *Gli2*$^{+/Fl}$; *Gli3*$^{Fl/Fl}$	x	*Gli2*$^{Fl/Fl}$;*Gli3*$^{Fl/Fl}$;Rosa$^{Gli3/Gli3}$	*Shh*$^{Cre/+}$; *Gli2*$^{Fl/Fl}$; *Gli3*$^{Fl/Fl}$;Rosa$^{Gli3/+}$	ZPA-Gli2/3KO;Tg-Gli3R+
Shh$^{Cre/+}$; *Gli2*$^{+/Fl}$; *Gli3*$^{Fl/Fl}$	x	*Gli2*$^{Gli1/Fl}$; *Gli3*$^{Fl/Fl}$	*Shh*$^{Cre/+}$; *Gli2*$^{Gli1/Fl}$; *Gli3*$^{Fl/Fl}$	ZPA-Gli2/3KO;Gli1KI+
Shh$^{Cre/+}$; *Smo*$^{+/Fl}$	x	*Smo*$^{Fl/Fl}$	*Shh*$^{Cre/+}$; *Smo*$^{Fl/Fl}$	ZPA-SmoKO
Shh$^{Cre/+}$; *Bmpr1a*$^{+/FL}$	x	*Shh*$^{-/+}$; *Bmpr1a*$^{FL/FL}$	*Shh*$^{Cre/+}$; *Bmpr1a*$^{FL/FL}$	ZPA-Bmpr1aKO
*Msx2*Cre	x	*Rosa*$^{SmoM2/ SmoM2}$	*Msx2*Cre; Rosa$^{SmoM2/+}$	AER-SmoM2
*Msx2*Cre;*Ptch1*$^{+/ Fl}$	x	*Ptch1*$^{Fl/Fl}$	*Msx2*Cre; *Ptch1*$^{Fl/Fl}$	AER-Ptch1KO
*Msx2*Cre; *Gli2*$^{FL/Fl}$; *Gli3*$^{Fl/Fl}$	x	*Gli2*$^{Fl/Fl}$; *Gli3*$^{Fl/Fl}$	*Msx2*Cre; *Gli2*$^{FL/Fl}$; *Gli3*$^{Fl/Fl}$	AER-Gli2/3KO
*Msx2*Cre; *Smo*$^{+/Fl}$	x	*Smo*$^{Fl/Fl}$	*Msx2*Cre; Smo$^{FL/FL}$	AER-SmoKO
*Msx2*Cre; *Bmpr1a*$^{+/\Delta}$	x	*Bmpr1a*$^{FL/FL}$	*Msx2*Cre; *Bmpr1a*$^{FL/\Delta}$	AER-Bmpr1aKO
*Msx2*Cre;*Shh*$^{Cre/+}$;*Bmpr1a*$^{+/\Delta}$	x	*Bmpr1a*$^{FL/FL}$	*Msx2*Cre; *Bmpr1a*$^{FL/\Delta}$	ZPA/AER-Bmpr1aKO
Sp8$^{CreER/+}$; *Bmpr1a*$^{FL/FL}$	x	*Bmpr1a*$^{FL/FL}$	*Sp8*$^{CreER/+}$;*Bmpr1a*$^{FL/FL}$	Sp8CreER-AER-Bmpr1aKO
Sp8$^{CreER/+}$; *Bmpr1a*$^{FL/FL}$	x	*Shh*$^{Cre/+}$;*Bmpr1a*$^{+/FL}$	*Shh*$^{Cre/+}$;*Sp8*$^{CreER/+}$;*Bmpr1a*$^{FL/FL}$	ZPA/Sp8creER-Bmpr1aKO
Shh$^{+/-}$;*Gli3*$^{+/Xt-J}$	x	*Shh*$^{+/-}$;*Gli3*$^{+/Xt-J}$	*Shh*$^{-/-}$;*Gli3*$^{Xt-J/Xt-J}$	† *Shh*$^{-/-}$;*Gli3*$^{-/-}$

†*Gli3*$^{+/Xt-J}$ is a *Gli3* null allele.
